# Research Progress of Small Plant Peptides on the Regulation of Plant Growth, Development, and Abiotic Stress

**DOI:** 10.3390/ijms25074114

**Published:** 2024-04-08

**Authors:** Guocheng Ren, Yanling Zhang, Zengting Chen, Xin Xue, Hai Fan

**Affiliations:** 1 Shandong Provincial Key Laboratory of Plant Stress, College of Life Sciences, Shandong Normal University, Jinan 250014, China; gcren5054@163.com (G.R.); 19862189693@163.com (Y.Z.); a19588936679@163.com (Z.C.); xuexin17865276262@163.com (X.X.); 2 Dongying Key Laboratory of Salt Tolerance Mechanism and Application of Halophytes, Dongying Institute, Shandong Normal University, No. 2 Kangyang Road, Dongying 257000, China

**Keywords:** small peptides, hormones, plant, abiotic stress

## Abstract

Small peptides in plants are typically characterized as being shorter than 120 amino acids, with their biologically active variants comprising fewer than 20 amino acids. These peptides are instrumental in regulating plant growth, development, and physiological processes, even at minimal concentrations. They play a critical role in long-distance signal transduction within plants and act as primary responders to a range of stress conditions, including salinity, alkalinity, drought, high temperatures, and cold. This review highlights the crucial roles of various small peptides in plant growth and development, plant resistance to abiotic stress, and their involvement in long-distance transport. Furthermore, it elaborates their roles in the regulation of plant hormone biosynthesis. Special emphasis is given to the functions and mechanisms of small peptides in plants responding to abiotic stress conditions, aiming to provide valuable insights for researchers working on the comprehensive study and practical application of small peptides.

## 1. Introduction

Based on their N-terminal signal sequences, small peptides are classified into secretory and non-secretory types. Secretory peptides include post-translationally modified peptides (PTMs) and cysteine-rich peptides (CRPs). The majority of them are secreted into the extracellular matrix through an endoplasmic-reticulum-dependent pathway, contributing to the regulation of extracellular growth and developmental activities [1]. Sometimes, they have an effect on the entire plant, functioning throughout the whole plant. Non-secretory peptides, characterized by their encoding through short open reading frames, differ significantly from secretory peptides. Non-secretory peptides work inside cells and often participate in cellular defense mechanisms. Since the identification of the first small plant peptide, systemin, a diverse array of peptide hormones has been discovered. These include plant sulfonamide (PSK) [2], C-terminally encoded peptides (CEP), CLAVATA3/Embryo Surrounding Region-related peptides (CLE), Inflorescence Deficient in Abscission (IDA), Rapid Alkalinization Factor (RALF), and Root Growth Factor (RGF), among others. These small peptides play a crucial role in the physiological activities of plants, encompassing cell communication [3], root development, microtubule organization, meristem maintenance, floral organ abscission, leaf senescence, nutrient assimilation, responses to diseases and pests, and fertilization. It is noteworthy that peptides also play an indispensable role in enabling plant responses to abiotic stressors such as salinity, drought, and extreme temperatures. This underscores the vital contribution of peptides to the adaptability and resilience of plants in diverse environmental conditions.

In the past, it was generally believed that small peptides were only involved in short-distance signal transduction in plants. However, recent research has shown that an increasing number of small peptides also play an important role in long-distance signal transduction [4]. Knowledge about the long-distance transport of peptides in response to abiotic stress, however, remains limited. The long-distance signal transduction of small peptides is accomplished through the peptide receptor system. Roots detect changes in the external environment, such as elevated salt levels or drought conditions, triggering the production of peptides. These peptides are then transported via the xylem to the stems, where they engage with specific receptors. Then, through a series of reactions, the plants’ tolerance to the environment is improved.

Despite notable advances in the study of small peptides, significant hurdles remain. Among these, a primary challenge is deciphering the signal transduction mechanisms between the upstream and downstream components of the signaling networks established by small peptides and their receptors. In this paper, we provide a comprehensive review of the diverse roles of small peptides in plant growth, development, and responses to abiotic stress. We delve into the involvement of small peptides in long-distance signaling pathways and examine their pivotal functions in the regulation of plant hormone biosynthesis.

## 2. Types and Sources of Small Peptides in Plants

The small peptides are commonly classified into three categories: cysteine-rich peptides (CRP), post-translationally modified peptides (PTM), and non-secretory small peptides [5,6,7,8,9] (Table 1). PTM and CRP, collectively referred to as secretory small peptides, share common characteristics, including N-terminal secretion signals and a central variable region. Peptides rich in cysteine and post-translationally modified peptides differ significantly in their origin, synthesis, and function. The formation of PTMs requires subsequent processing of proteins that have already been synthesized, while CRPs are formed directly after being encoded by genomic DNA and do not require post-translational modification. Mature PTM and CRP are produced through enzyme-mediated processing and modification of their precursor peptides [10].

### 2.1. Post-Translationally Modified Peptides

The process of post-translationally modified peptides involves tyrosine sulfation, proline hydroxylation, or arabinosylation, ultimately resulting in the formation of biologically active small peptides consisting of approximately 10 amino acid residues [29]. Tyrosine sulfation involves adding a sulfate group to the tyrosine residue, proline hydroxylation adds a hydroxyl group to the proline residue, and arabinosylation adds an arabinose molecule to the amino acid residue of a protein. Most of these small peptides are secreted into the extracellular space predominantly via passive diffusion [30]. Typical examples of post-translationally modified peptides include phytosulfokine (PSK), Tracheary Element Differentiation Inhibitory Factor (TDIF), CLAVATA3/EMBRYO Surrounding Region-Related (CLE), C-terminally Encoded Peptide (CEP), and Plant Peptide Containing Sulfated Tyrosine 1 (PSY) [23]. Since the discovery of the CLV3 gene, many other CLE genes have been successively identified. CLE peptides include A-type CLE peptides that promote root and shoot development, such as CLAVATA 3 (CLV3), which is involved in apical meristem differentiation, and B-type CLE peptides such as TDIF [13,14,31]. CLE peptides are mainly produced through post-translational arabinoxylan modification of precursor peptides. Their receptors are primarily leucine-rich repeat receptor-like kinases (LRR/RLKs), including CLAVATA1 (CLV1), CLAVATA2 (CLV2), CORYNE (CRN) [32], Receptor-Like Protein Kinase 2 (BAM) [33], HAESA-LIKE 1 (HSL1), and Somatic Embryogenesis Receptor-Like Kinase (SKM) [34]. The WUSCHEL (WUS) gene can induce the expression of CLV3, and CLV3 acts on CLV1 to inhibit the activity of WUS, establishing a negative feedback loop essential for the maintenance of shoot apical meristem (SAM) homeostasis [8,35,36]. In addition to interacting with the receptor CLV1, CLV3 also interacts with other receptors such as CLV2, CRN, receptor-like protein kinase 2 (RPK2) [37], and auxiliary receptors like somatic embryogenesis receptor kinase (SERK) [38]. PSK, another post-translationally modified peptide, becomes biologically active through tyrosine sulfation of its precursor [39]. Initially, PSK was isolated and identified from *Asparagus officinalis* L.

### 2.2. Cysteine-Rich Peptides

CRPs are distinguished by their multiple disulfide bonds and are named accordingly. The N-terminus of CRPs typically have a conserved signal peptide, while the C-terminus possesses an even number of cysteine residues, enabling the formation of disulfide bonds. Examples of CRPs include the RALF with four cysteine residues, late embryogenesis abundant-like protein 2-regulated (LURE), taximin (TAX) [24], lilly anther protein 2 (LAT52) [25], the epidermal patterning factor (EPF), and inflorescence deficient in abscission (IDA), among others. RALFs represent a category of cysteine-rich peptides derived from precursor peptides through cleavage by the S1P protein [22]. They are characterized by having four cysteine amino acid residues and are implicated in a variety of biological processes, including root development, cell wall remodeling, maintaining pollen tube integrity, facilitating fertilization, mediating immune responses, and modulating plant responses to environmental stress [40,41,42,43,44]. Furthermore, the discovery of new cysteine-rich peptides continues to expand our understanding of plant biology. For instance, a novel RALF-like peptide, EaF82, was recently identified and has been shown to reduce pollen and seed production [45].

### 2.3. The Relationship between Non-Secretory Small Peptides, Post-Translationally Modified Peptides, and Cysteine-Rich Peptides

Both PTMs and CRPs significantly contribute to the growth and developmental processes of plants. For instance, CLE40p activates CLV1 on the membrane, leading to the rapid phosphorylation of Cyclic Nucleotide-Gated Channel 9 and Cyclic Nucleotide-Gated Channel 6. This results in an elevated cytoplasmic Ca^2+^ concentration within the proximal meristematic tissue, thereby regulating the differentiation of the proximal root meristem [46]. Similarly, TAX, a membrane-localized peptide, plays a crucial role in regulating plant-specific metabolic pathways. When combined with methyl jasmonate (MeJA), TAX notably increases paclitaxel production and enhances the biosynthesis of alkaloids in tobacco roots [24].

Current research on non-secretory small peptides remains limited, leading to a relatively superficial comprehension of their interaction with secretory small peptides within signal transduction pathways. Nevertheless, recent discoveries indicate that in Arabidopsis, the non-secretory small peptide rotundifolia 4 (ROT4) enhances its interaction with brassinosteroid-signaling kinase 5 (BSK5) through S-acylation. This heightened interaction disrupts the binding of receptor kinase PEP receptor 1 (PEPR1) to BSK5, which is crucial for signaling of the secretory peptide plant elicitor peptide 1 (PEP1), thereby playing a significant role in regulating plant immunity [11]. This implies that secretory and non-secretory peptides do not act independently but interact with each other to collectively regulate plant growth and development processes. Non-secretory small peptides may serve as “switches”, modulating the activities of secretory small peptides and, thus, maintaining a dynamic equilibrium within the plant’s physiological processes. Such research sheds light on the possible regulatory roles of non-secretory small peptides in plant signal transduction and opens new avenues for understanding the complexity of plant physiological mechanisms.

## 3. The Role of Small Peptides in Plant Growth and Development

Small plant peptides play pivotal roles in regulating a diverse range of growth and developmental processes in plants. These roles encompass facilitating intercellular communication, promoting root development, influencing microtubule formation, maintaining meristem activity, orchestrating floral organ abscission, driving leaf senescence, optimizing nutrient uptake, and supporting fertilization, among others.

### 3.1. The Role of Small Peptides in Root Development

Small plant peptides are vital to multiple facets of plant root development, impacting virtually every phase of root formation. They play a significant role in nodulation, primary root growth, and the initiation of lateral roots. Furthermore, small plant peptides also influence the division, elongation, and differentiation of root apex cells [47,48].

Recent research has indicated that nodulation can promote plant flowering, thereby linking nodulation with various stages of plant growth and development [49]. Peptides such as CLE [50,51], CEP [16], and microRNA-encoded peptides (miPEPs) have been identified as key regulators of plant root nodulation [17]. It has been found that under conditions of ample soil nitrogen, CLE peptides produced in the roots transport over long distances to the stem, where they interact with the peptide receptor-like kinase HAR1, resulting in the suppression of miR2111 expression and, thus, controlling the number of root nodules in roots [52]. In contrast, under low-nitrogen conditions, CEP signaling peptides can trigger the production of miR2111, facilitating nodule formation even in the absence of root nodule bacteria [53]. These findings suggest a complex negative feedback loop between CLE and CEP peptides, which plays a crucial role in modulating root nodule formation. Nonetheless, the specific interactions between CLE and CEP peptides in the regulation of root nodulation warrant further investigation.

Small plant peptides fulfill diverse roles in regulating root growth, with specific peptides influencing root development in distinct ways. For instance, small plant peptides might contribute to root development and adaptation to flooding [54]. 

The regulatory role of CEP in root growth has been investigated in cucumber, Arabidopsis, *Brassica rapa* L., and other plants. For example, knocking out the CEP gene in Arabidopsis resulted in increased primary root length and number of lateral roots [55,56]. Similarly, peptides such as BrCEP3 and BrCEP26 have been demonstrated to significantly enhance primary root growth in moss [57]. 

**Figure 1:**
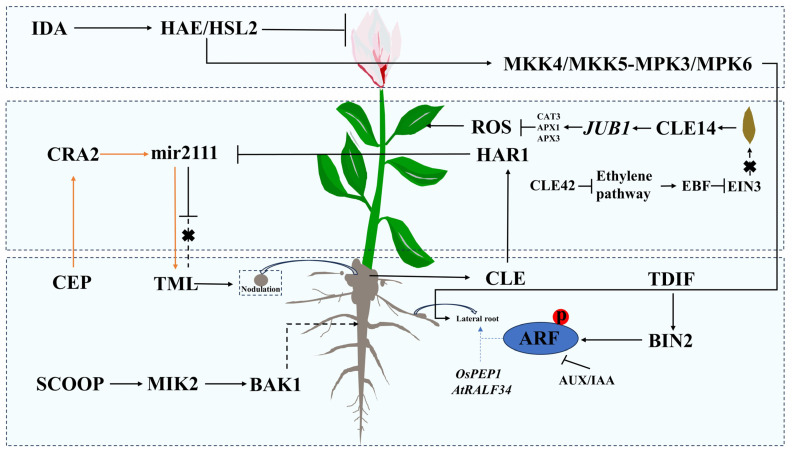
The function of small plant peptide in regulating growth and development. Small plant peptides exert different effects in various parts of the plant, including roots, stems, leaves, and flowers. In root regulation, both CLE and CEP can be transported from the roots to the stem, acting on mir2111. CLE has an inhibitory effect on root nodules, while CEP promotes the formation of plant root nodules. TDIF positively regulates lateral root development by promoting the phosphorylation of ARF receptors. Additionally, the peptides OsPEP1 and RALF34 have promoting effects on lateral root development in rice and in Arabidopsis, respectively. SCOOP stimulates root growth by interacting with MIK2, leading to the formation of a co-receptor complex with BAK1. In leaf regulation, CLE14 delays leaf senescence by inhibiting the expression of genes related to reactive oxygen species (ROS) synthesis, while CLE42 slows down leaf senescence by closing the ethylene synthesis pathway. Regarding flower organ regulation, IDA inhibits the abscission of flower organs by acting on the receptor HAE/HSL2. The arrow and T-line without arrowheads respectively represent positive and negative regulation in the signaling pathway. Solid lines indicate direct regulation, while dashed lines indicate the presence of multiple steps or unknown components. Cross mark represents suppression.

In grapevines, the peptide vvi-miPEP171d has been identified as a regulator of adventitious root formation [58]. In rice, overexpression of *OsPEP1* results in a shorter root phenotype, highlighting PEP1’s regulatory role in root development (Figure 1). The root meristem growth factor (RGF), a 13-amino acid sulfated peptide, contributes to the maintenance of root apical meristem activity [18]. However, the specific action site of RGF at the root apex and its regulation of PLETHORA proteins are yet to be clarified. 

Serine Rich Endogenous Peptides (SCOOPs), encoded by the Brassicaceae-specific PROSCOOP family, are commonly believed to participate in defense signal transduction when plants are subjected to insect attacks or mechanical damage [26]. Recent research indicates that SCOOP12 regulates defense responses and root elongation in Arabidopsis. SCOOP12 reduces the size of the meristem and cell length in the root, suggesting a role in shortening the root under specific conditions. SCOOPs are induced when plants are invaded by a pathogen or microbe and act as a mediator of intermediate filament formation kinase 2 (MIK2), forming a co-receptor with BRI1-associated receptor kinase 1 (BAK1) to regulate downstream signaling pathways (Figure 1) [27]. 

Furthermore, small peptides like TDIF are key to lateral root development, binding to their receptor TDIF initiated (TOR) and activating BIN, which in turn promotes the phosphorylation of AUX/IAA regulatory genes to facilitate lateral root development (Figure 1). *AtRALF34* is considered to be part of the gene regulatory network for lateral root initiation in Arabidopsis. In contrast to *AtRALF34*, its homologous gene *CsRALF34* in cucumber does not participate in lateral root initiation but instead leads to G2/mitosis transition block [59,60,61].

In rice, the cleavage inducing factors OsCIF1 and OsCIF2 play a role in forming Casparian Strips in both endodermal and non-endodermal cell layers of the root, illustrating peptides’ regulatory capacity on root pericycle development as well [15].

### 3.2. The Role of Small Peptides in Leaf Senescence 

In addition to their role in root development, peptides also influence leaf senescence. Studies have shown that the small peptide CLE14 is involved in regulating plant leaf senescence [62]. Treating detached Arabidopsis leaves with CLE14 peptide significantly reduces senescence. When plants undergo stress-induced leaf senescence or age-induced leaf senescence, CLE14 targets JUB1 to enhance the expression of genes involved in the removal of reactive oxygen species (ROS), thus mitigating leaf senescence (Figure 1). This study also indirectly indicates the role of CLE peptides in long-distance signal transduction from roots to leaves. Furthermore, the study found that overexpression and exogenous application of the peptide CLE42 can also delay leaf senescence. CLE42 counters the ethylene pathway by inhibiting ethylene biosynthesis, increasing levels of the ethylene-insensitive 3 (EIN3)-binding F-box protein (EBF) and reducing the activity of ethylene-related transcription factor EIN3 (Figure 1) [63]. However, the redundancy in CLE peptide functions, such as simultaneous expression of CLE1 and CLE4 in identical tissues, presents challenges in fully understanding their roles [64]. The involvement of CLE peptides in leaf processes underscores their potential as long-distance signaling molecules. 

### 3.3. Other Functions of Small Peptides in Plant Growth and Development

In addition to the aforementioned functions, peptides significantly contribute to various aspects of plant physiology, including nutrient uptake, circadian rhythms, photomorphogenesis, resistance against pests and diseases, as well as the regulation of plant immunity [65,66,67,68]. For instance, synthetic CEP peptides have been shown to improve the uptake of nitrates, phosphates, and sulfates in plant roots [69]. The regulation of CLE peptide gene expression is influenced by the phosphorus and sulfur statuses in Arabidopsis. The antimicrobial peptide DmAMP1W from wheat curtails the proliferation of fungi, including *fungi Rhizoctonia cerealis* and *Bipolaris sorokiniana*, effectively combating wheat sharp eyespot and root rot diseases [70]. Additionally, a variety of small peptides, such as those released by papain-like cysteine proteases, Defensin-Derived Antifungal Peptides, and small cysteine-rich (SCR) secreted proteins like RsMf8HN, play an integral role in plants’ defense against fungal and bacterial pathogens [71,72,73]. The peptide VISP1, acting as a selective autophagy receptor, plays an essential role in plant immunity [74]. The external application of RALFs peptide holds promise for surmounting interspecific hybridization barriers within the Brassicaceae family [75]. Peptides are also pivotal in controlling flower organ abscission. By using CRISPR/Cas technology to simultaneously knock out the *BnaIDA-A07* and *BnaIDA-C06* genes in rapeseed, the shedding of floral organs is effectively inhibited [19].

In summary, peptides exhibit diverse functions across various plant parts, including roots, stems, leaves, flowers, and more.

## 4. The Role of Small Peptides in Plant under Abiotic Stress 

The global warming of the climate and salinization pose significant constraints on the development of agricultural production. These conditions significantly constrain agricultural development and plant growth. Unlike animals, plants cannot freely move, rendering them vulnerable to various environmental stressors including temperature extremes, water scarcity, and saline–alkali conditions, which severely impact their growth and development. In coping with these challenges, small peptides, acting as signaling molecules, play a crucial role in plants’ response to abiotic stress [76].

### 4.1. The Role of Small Plant Peptides under Drought Stress

**Figure 2:**
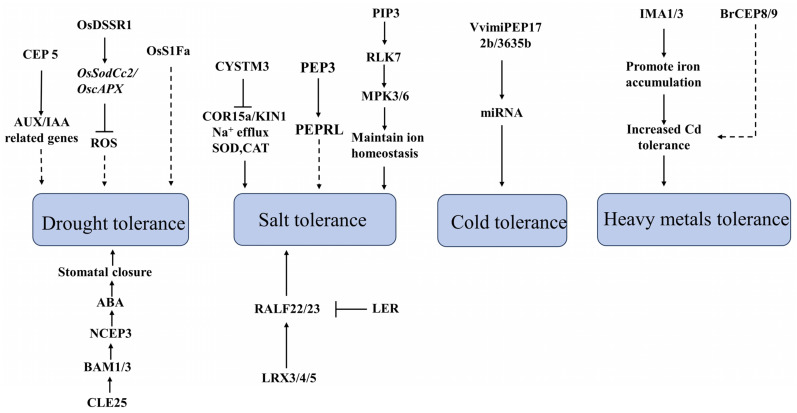
Related signal pathways of small peptides in drought, salt, temperature, and heavy metals stress. The arrow and T-line without arrowheads respectively represent positive and negative regulation in the signaling pathway. Solid lines indicate direct regulation, while dashed lines indicate the presence of multiple steps or unknown components.

In recent years, the escalation of drought conditions globally has markedly affected plant growth, leading to substantial reductions in crop yields. The adverse effects of drought on plant life are comparable to those caused by pathogens. Extensive research has demonstrated the critical role of small peptides in plants’ responses to drought stress. Researchers have discovered a number of small peptides that assist plants in resisting drought stress. Specifically, the overexpression of *OSS1Fa1* from rice in Arabidopsis substantially enhances the drought tolerance of Arabidopsis (Figure 2). Furthermore, S1Fa has been shown to bolster the tolerance of various other plants, including corn and Chinese cabbage, to a range of abiotic stresses. Nevertheless, a comprehensive understanding of the biochemical, molecular, and physiological functions of S1Fa remains elusive. Further investigation of its interacting proteins is essential to unravel its contribution to plant stress resistance [77]. In rice, a novel signaling peptide, OsDSSR1, has been discovered, triggering the expression of ROS scavenging-related genes, *OsSodCc2* and *OscAPX*, under drought conditions (Figure 2). This peptide boosts the activity of superoxide dismutase (SOD) and ascorbate peroxidase (APX), facilitating ROS clearance and enhancing rice’s drought tolerance [78]. Additionally, OsRALF45/46 in rice interacts with the receptor-like kinase OsMRLK63, leading to improved drought resistance [79]. 

Research on CEP regulation of plant drought tolerance has been extensive, and studies in crops such as wheat and tomato have revealed the crucial role of CEP in enhancing plant drought tolerance [80]. Application of the TaCEP1D peptide on wheat leaves has been shown to improve drought resistance [81]. Some researchers have found that the receptors of CEP peptides are CEPR1 and CEPR; these two, along with RLK7, share high similarity in protein sequences and belong to the same LRR-RLK subfamily XI. Therefore, the CEP peptides involved in wheat drought and salt stress tolerance may also rely on RLK7, and further research is needed to elucidate the receptors and mechanisms of action. 

CLE25, synthesized in the roots, targets membrane receptors BAM1 and BAM3 in the shoots. This interaction stimulates the expression of *NCED3* and promotes ABA production, subsequently inducing stomatal closure in response to drought stress (Figure 2) [82,83]. This elucidates the capability of the CLE25 peptide to regulate plant growth and development through systemic signaling. 

The Small Paraquat resistance protein (SPQ), LcSPQ, equipped with an N-terminal signal peptide, has been found to enhance plant drought resistance upon overexpression [84]; however, its specific receptors and underlying mechanisms require additional study. 

Recent research has expanded the investigation of PSK (phytosulfokine) responses to stress from model organisms, such as Arabidopsis, to staple crops and even tall trees. After conducting a bioinformatics analysis of the rice PSK family, some researchers found that when plants are subjected to heat, salt, drought, and cold stress, the *OsPSKR* gene functions to enhance the plant’s resistance to various adverse environmental conditions [85]. The action of PSK under stress conditions is mediated by various subtilisin-like proteases of the phytaspase subtype, which cleave the PSK precursor, enabling its functionality under diverse stress conditions. In Arabidopsis, the subtilisin-like protease SBT3.8 cleaves the C-terminal end of the PSK precursor, enhancing drought resistance [2,39]. In tomato, phytaspase 2 processes the aspartic acid in the PSK precursor, facilitating flower and fruit abscission under drought stress [86,87]. PSK has also been shown to contribute to ROS homeostasis by modulating the activity of peroxidase in Cunninghamia lanceolata [83]. Current research on PSK precursor processing has predominantly focused on the C-terminal end, with further studies needed to explore the processing at the N-terminal end.

### 4.2. The Role of Small Peptides under Salt Stress in Plants

Globally, salinization affects over 10% of land, significantly hindering agricultural productivity. Salt stress triggers ion imbalance, osmotic shifts, oxidative stress, and secondary stresses in plants [88]. 

The peptide PIP3, activated by Pathogen-Associated Molecular Patterns (PAMPs), targets the receptor-like kinase 7 (RLK7), characterized by leucine-rich repeat motifs, under salt conditions (Figure 2). This activation of RLK7 enhances its activity, resulting in the amplification of salt stress signals by activating MPK3/6, maintaining cellular ion homeostasis, and thereby enhancing plant salt tolerance [20].

Additionally, AtPEP3, a hormone-like peptide produced by the *AtPROPEP3* gene, augments plant salt tolerance through its interaction with the PEPR1 receptor (Figure 2). When plants experience salt stress, exacerbating cell damage leads to the release of AtPEP3, which in turn induces downstream signaling responses. However, the intricacies of AtPEP3’s signal transduction pathway in salt tolerance remain elusive. While pathogen-induced AtPEP3 acts on both PEPR1 and PEPR2 receptors, salt-induced AtPEP3 specifically targets PEPR1, hinting at potential salt-induced conformational changes in PEPR2 that require further investigation [89].

The RALF-FER pathway plays a pivotal role in plant stress adaptation (Figure 2) [90]. Under normal conditions, LRX3/4/5 proteins, which contribute to plant growth and cell wall integrity, bind RALF22/23 peptides. This interaction prevents RALF peptides from binding to FER proteins, thus inhibiting FER internalization (Figure 2). In contrast, under salt stress, LRX3/4/5 proteins perceive changes in the cell wall, leading to the dissociation of RALF peptides from LRX. This promotes the binding of RALF22/23 peptides to the FER protein, resulting in the loss of its salt tolerance capability and the manifestation of a salt-sensitive phenotype [91]. 

Research on CYSTM (Cys-rich Peptide of the Seed Tryptic Mixture) peptides, particularly regarding stress responses, is nascent, with a focus on bioinformatics analyses. CYSTM, identified as a novel cysteine-rich transmembrane module, plays a role in stress tolerance across eukaryotes [28]. CYSTM can modulate the plant’s tolerance to heat and ultraviolet stress. Additionally, CYSTM3 negatively regulates Arabidopsis’ salt stress tolerance [92]. Notably, CYSTM3 has been shown to diminish salt tolerance in Arabidopsis by impeding Na^+^ efflux and inhibiting ROS-scavenging enzymes, alongside downregulating salt-responsive gene expression (Figure 2) [93]. Given its mitochondrial localization, further research is required to elucidate CYSTM3’s impact on Ca^2+^ ion homeostasis and plant salt tolerance. The relationship between cysteine residues and transmembrane helices in CYSTM peptides also remains to be explored.

### 4.3. The Role of Small Plant Peptides under Temperature Stress

The fluctuation in temperature exerts a significant influence on plants, yet the role of small peptides in modulating temperature stress responses remains underexplored. It is established that low-temperature stress alters membrane composition, reactive oxygen species (ROS) levels, osmoregulation substances levels, and antioxidant capacities [94,95]. Beyond their known functions in growth and development, microprotein-encoded peptides (miPEPs) also contribute to abiotic stress resilience. Specifically, under cold conditions, plants produce miPEPs encoded by pri-miRNAs, such as vvi-miPEP172b and vvi-miPEP3635b (Figure 2). Studies have demonstrated that these peptides bolster plant cold resistance by modulating miRNA gene activity [96]. In contrast, during high-temperature exposure, the CLE45 peptide, which originates in the stigma and extends to the transmitting tract, suggests a potential function in heat stress adaptation [97]. 

### 4.4. The Role of Small Peptides under Other Stress Conditions and Biological Stress in Plants

Peptides play a pivotal role in augmenting plant tolerance to heavy metals. The long-distance signaling peptide IMA, which mediates root-to-shoot communication, is instrumental in this context. Specifically, IMA1 and IMA3 have been shown to enhance tolerance to cadmium (Cd) toxicity through the promotion of iron (Fe) accumulation (Figure 2) [98]. Furthermore, exposure to cadmium triggers an increase in the expression of *BrCEP8* and *BrCEP19*, indicating that *BrCEPs* may facilitate cadmium absorption in Brassicaceae, thereby augmenting Brassicaceae’s tolerance to elevated Cd levels (Figure 2) [57]. Additionally, microRNA-encoded peptides (miPEPs) have been recognized for their regulatory role in plant tolerance to cadmium [99].

Additionally, under conditions of salt stress, PAMP-induced secreted peptides (PIP3) target receptor-like kinase 7 (RLK7), enhancing salt tolerance [20]. RLKs are pivotal in mediating plant stress resistance, with IDA peptides acting on receptors within the RLK family, hinting at IDA’s potential involvement in plant stress resilience. Recent research has unveiled that IDA family members, specifically IDL6 and IDL7, play a contrasting role by negatively affecting stress resistance in Arabidopsis through the acceleration of reactive oxygen species (ROS) production [100].

Small peptides play a regulatory role in modulating immune responses within plants [101]. Moreover, research has revealed that under phosphate-deficient conditions, PHR1 activates the RALF peptide, which in turn triggers the FERONIA receptor. This activation sequence suppresses pathogen-induced immunity, thereby facilitating the proliferation and development of root microbiota. Consequently, this mechanism contributes to mitigating the effects of phosphate deficiency in plants [102]. ROOT MERISTEM GROWTH FACTOR 7 is regulated by perception through the RGI4/RGI5-BAK1/SERK4 receptor complex in the immune response of Arabidopsis [103]. In addition, virus-induced small peptide 1 (VISP1) [104] and the small peptide StPIP1 in potato [105] also participate in the immune response of plants.

Under phosphate scarcity, Arabidopsis PHR1 directly binds to and activates the promoter of the Rapid Alkalinization Factor (RALF) gene. Subsequently, RALF inhibits the formation of the immune receptor complex triggered by pathogen-associated molecular patterns (PAMPs), via FERONIA. This PHR1-RALF-FERONIA pathway dampens immune responses, facilitating specific root microbiota colonization. This colonization aids in mitigating phosphate starvation by boosting the expression of phosphate starvation response (PSR) genes.

## 5. Long-Distance Signal Transduction Mediated by Small Peptides under Stress

Small peptides can function as both local signaling molecules in roots and long-distance signals, acting on receptors in various plant tissues [4]. The presence of peptides such as CLE, CEP, and CIF in tomato xylem sap highlights their role in root-to-shoot signal transmission [106]. Furthermore, recent discoveries of CEP and CLE peptides in the phloem suggest their involvement in shoot-to-root signaling [107]. The PSY family’s participation in root-to-shoot transport supports the potential for investigating sulfated peptides’ responses to drought stress [21]. During stress conditions, these peptides regulate the plant’s physiological state as long-distance signals. For instance, under drought, Arabidopsis CLE25, produced in the roots, travels to the leaves to activate the BAM1/3 receptor, triggering the synthesis of abscisic acid by upregulating *NINE-CIS-EPOXYCAROTENOID DIOXYGENASE 3* (*NCED3*) [83]. Similarly, CEP peptides mediate nitrogen demand and support root nodule and lateral root formation. Nitrogen scarcity prompts CEP expression in roots, with the signal reaching the stem and activating LRR-RKs [3]. Beyond CLE, advancements in understanding long-distance signaling have been underscored by grafting experiments, which demonstrate the necessity of CEPR1 activity in both roots and shoots for modulating lateral root growth [108,109]. The comprehensive effects of CEP peptides on plant parts like leaves, shoots, and roots via long-distance transport are areas for ongoing research.

## 6. Plant Peptides Control the Biosynthesis of Hormones

The discovery of peptides involved in long-distance signal transduction has underscored their similar regulatory pathways to those of hormones. Hormones are known to facilitate plant growth and development through diverse interactions, highlighting the emerging interest in peptides’ regulatory roles within these hormone interactions among the scientific community. 

The overexpression of *RsCLE22a*, for instance, elevates auxin levels in *Raphanus sativus* L., consequently inhibiting the growth of the primary root [71]. FER (FERONIA) is regulated by various hormones, such as ABA, ethylene, and jasmonic acid. For example, treating apple (*Malus domestica*) roots with ABA significantly induces the expression of *MdFER* [110,111]. Furthermore, overexpressing *MdFER* in apples can negatively regulate their sensitivity to ABA. Ethylene can also promote the transcription of FER in the roots. Due to the interaction between RALF and FER under salt stress, we speculate that RALF may also have intricate connections with plant hormones, akin to other plant peptides [112]. Recent findings indicate that ethylene downregulates the expression of *CsRALF34* in cucumber [71], whereas in Arabidopsis, *AtRALF34* is modulated by both auxin and ethylene [59]. Furthermore, the external application of SpPIP1 modulates jasmonic acid levels, thereby enhancing tomato resistance to stress [113]. *GhRALF1* influences cotton’s diurnal rhythms and fiber development through modulating auxin signaling and proton pump activity [114]. 

Notably, auxin accumulation in the root system has been associated with increased wheat yields under drought conditions. Drought-responsive transcription factors regulate auxin signaling by increasing AUX/IAA expression, and CEP5 signaling has been shown to stabilize the AUX/IAA transcriptional repressor [115]. DEVIL-like (DVL) peptides play a pivotal role in sustaining root growth under abiotic stress conditions by affecting ABA-related gene expression [116]. The synergistic interaction between ABA-INDUCED EXPRESSION 1 (AIN1) and PIP2 significantly bolsters plant drought resistance, underscoring the critical role of ABA [117]. CEP peptides indirectly regulate plant responses to cytokinins by affecting components within the cytokinin signaling pathway [118]. Finally, the TDIF peptide targets auxin-related genes, altering internal auxin concentrations and, thus, orchestrating the growth of both primary and lateral roots [12].

In conclusion, peptides significantly affect plant growth and development by modulating plant hormone concentrations. Importantly, a growing body of research identifies peptides as key regulators in plants’ responses to abiotic stresses, alongside crucial hormones such as ABA, which are known to play a pivotal role in plant stress resistance. Thus, investigating the effects of peptides on plant hormones is of paramount importance for a deeper understanding of how plants respond to abiotic stress.

## 7. Discussion and Prospects

Research on the diverse families of plant peptides is still in its infancy, partly due to the challenges associated with isolating these molecules. However, advancements in peptideomics are increasingly enabling the identification of new peptides. Techniques such as Capillary Electrophoresis (CE) and Microchip Electrophoresis (MCE) have improved the efficiency of peptide separation, addressing the research difficulties posed by their small size. Recent technological breakthroughs, including nanoscale liquid chromatography-tandem mass spectrometry (nano-LC–MS/MS), alongside predictive tools like SignalP 6.0 and ExamPle, have facilitated the discovery and analysis of novel peptides [119,120,121]. To tackle functional redundancy, antagonistic peptide technology offers a promising solution [122], while CRISPR gene editing presents a way to obtain mutants for studying functional loss. Most small peptides undergo post-translational modifications, with a multitude of enzymes involved in their processing and modification. Further research is needed to understand how these enzymes influence the biological activity and signal transduction of peptides. Small peptides play crucial roles in various aspects of plant growth and development. Despite the critical signaling roles of peptides, many of their receptors remain unidentified, and the mechanisms of receptor perception and the role of auxiliary receptors in peptide signaling are still under-researched. The “one-step” nanoimprint method has emerged as a novel approach for receptor identification [120]. While exogenous peptide application is common in functional studies, differences from endogenous peptides due to processing and modifications are crucial considerations. Recent research utilizing isotope-labeled peptides has shed light on the effects of post-translational modifications, underscoring the complexity of peptide signaling pathways [123].

Through research, scientists have discovered that an increasing number of small peptides co-regulate plant growth and development with hormones. For example, there is a significant overlap in the signal transduction between CLE and hormones such as auxin, cytokinin, gibberellin, and abscisic acid [31]. Peptides are also known to collaborate with hormones in responding to environmental changes [97]. Although the hormonal aspects of plant stress responses are well-studied, the intricate regulatory network between peptides and plant hormones during such responses still demands further clarification. A notable gap in current research is a molecular-level understanding of how peptides respond to environmental stressors. For instance, the mechanism by which the rice *OSS1Fa1* gene enhances drought tolerance in Arabidopsis remains poorly defined, indicating a general shortfall in exploring the proteins that interact with these genes. Despite these challenges, significant progress has been made in certain areas, such as the study of Arabidopsis CLE14, which has been shown to respond to drought and salt stress [62]. This research discovered that CLE14 activates JUB1, leading to increased expression of genes involved in ROS clearance and delayed leaf senescence. Such findings are pivotal, advancing the research on CLE peptides from a focus on development to include stress response and shedding light on the relationships between CLE genes and their downstream effects.

In recent years, the study of peptide-mediated long-distance signal transduction has emerged as a significant area of research. For instance, it has been discovered that overexpressing CLE3 in Arabidopsis roots leads to its transport to the shoots via long-distance signaling, where it influences WRKY33, thereby modulating the plant’s immune responses [124]. Additionally, the detection of the root-derived sugar peptide CLE-RS in the plant xylem points to its potential role in facilitating long-distance communication [125]. However, despite being acknowledged, the dynamics of peptide signaling under stress conditions remain largely unexplored. This research gap underscores the need for further investigation into how peptides contribute to plant adaptation and survival under adverse environmental conditions.

The primary objective of researching plant peptides is to benefit agriculture and foster agricultural advancement. Notably, the use of synthetically produced peptides externally applied to plants has been shown to regulate growth, development, and immune responses effectively. This indicates that peptides have considerable potential to serve as alternatives to traditional pesticides and chemical fertilizer. By harnessing the regulatory capabilities of peptides, we can develop safer, more sustainable agricultural practices that reduce reliance on chemical pesticides and enhance crop resilience.

## Figures and Tables

**Table 1:** Different types of small peptides and their functions in plant.

Types	Small Peptides	Function	References
Non-secretory small peptides	early nodulin	root nodule development, intracellular localization of proteins, etc.	[5,6,7,8,9]
	ROT4	regulates plant immunity, modulates cell proliferation, etc.	[11]
Post-translationally modified peptides	PSK	abiotic stress, cell division, root development, etc.	[2]
	TDIF	tracheary element differentiation inhibitory factor, root development, etc.	[12]
	CLE	abiotic stress, root development, leaf senescence, etc.	[13]
	CLV3	differentiation of the apical meristem, etc.	[14]
	CIF	formation of the Casparian strip, etc.	[15]
	CEP	abiotic stress, root development, nodulation, etc.	[16]
	miPEP	abiotic stress, fruit ripening, flower development, photosynthesis, nodulation, etc.	[17]
	RGF	maintaining the activity of the root apical meristem, abiotic stress, etc.	[18]
	IDA	floral organ abscission, etc.	[19]
	PIP	abiotic stress, etc.	[20]
	PSY	abiotic stress, root development, etc.	[21]
Cysteine-rich peptides	RALFs	root development, cell wall remodeling, immune response, abiotic stress, etc.	[22]
	LURE	promoting pollen tube germination, etc.	[23]
	TAX	regulating plant-specific metabolism, etc.	[24]
	EPF	regulating epidermal cell patterns, etc.	[23]
	LAT52	pollen hydration process, pollen tube growth, etc.	[25]
	SCOOPs	root development, immune response, etc.	[26,27]
	CYSTM	abiotic stress, etc.	[28]

## Data Availability

Data will be made available on request.
